# Effect of Inorganic and Organic Carbon Enrichments (DIC and DOC) on the Photosynthesis and Calcification Rates of Two Calcifying Green Algae from a Caribbean Reef Lagoon

**DOI:** 10.1371/journal.pone.0160268

**Published:** 2016-08-03

**Authors:** Friedrich W. Meyer, Nadine Schubert, Karen Diele, Mirta Teichberg, Christian Wild, Susana Enríquez

**Affiliations:** 1 Leibniz Center for Tropical Marine Ecology (ZMT), Bremen, Germany; 2 Laboratory of Photobiology, Unidad Académica de Sistemas Arrecifales Puerto Morelos (ICMyL), Universidad Nacional Autónoma de México (UNAM), Cancún, Mexico; 3 Edinburgh Napier University, School of Life, Sport and Social Sciences, EH11 4BN Edinburgh, United Kingdom; 4 Faculty of Biology & Chemistry, University of Bremen, Bibliothekstraße 1, Bremen, Germany; 5 St Abbs Marine Station, St Abbs, Berwickshire, TD14 5QF, United Kingdom; Academia Sinica, TAIWAN

## Abstract

Coral reefs worldwide are affected by increasing dissolved inorganic carbon (DIC) and organic carbon (DOC) concentrations due to ocean acidification (OA) and coastal eutrophication. These two stressors can occur simultaneously, particularly in near-shore reef environments with increasing anthropogenic pressure. However, experimental studies on how elevated DIC and DOC interact are scarce and fundamental to understanding potential synergistic effects and foreseeing future changes in coral reef function. Using an open mesocosm experiment, the present study investigated the impact of elevated DIC (pH_NBS_: 8.2 and 7.8; pCO_2_: 377 and 1076 μatm) and DOC (added as 833 μmol L^-1^ of glucose) on calcification and photosynthesis rates of two common calcifying green algae, *Halimeda incrassata* and *Udotea flabellum*, in a shallow reef environment. Our results revealed that under elevated DIC, algal photosynthesis decreased similarly for both species, but calcification was more affected in *H*. *incrassata*, which also showed carbonate dissolution rates. Elevated DOC reduced photosynthesis and calcification rates in *H*. *incrassata*, while in *U*. *flabellum* photosynthesis was unaffected and thalus calcification was severely impaired. The combined treatment showed an antagonistic effect of elevated DIC and DOC on the photosynthesis and calcification rates of *H*. *incrassata*, and an additive effect in *U*. *flabellum*. We conclude that the dominant sand dweller *H*. *incrassata* is more negatively affected by both DIC and DOC enrichments, but that their impact could be mitigated when they occur simultaneously. In contrast, *U*. *flabellum* can be less affected in coastal eutrophic waters by elevated DIC, but its contribution to reef carbonate sediment production could be further reduced. Accordingly, while the capacity of environmental eutrophication to exacerbate the impact of OA on algal-derived carbonate sand production seems to be species-specific, significant reductions can be expected under future OA scenarios, with important consequences for beach erosion and coastal sediment dynamics.

## Introduction

The rise of oceanic *p*CO_2_ caused by increasing CO_2_ concentrations in the atmosphere is leading to significant changes in the ocean carbonate system, which are primarily reflected in an increase in bicarbonate concentration and a decrease in seawater pH (ocean acidification- OA) [1, 2]. These changes also induce a significant decline in the saturation state of the different crystallization forms of calcium carbonate in the marine environment, which will facilitate the dissolution of existing calcium carbonate deposits and cause severe impacts on marine calcifiers. Many coral reef habitats and their lagoons are particularly threatened by ocean acidification. Studies conducted at natural low pH sites have shown that under OA the reef framework is less stable [3], and reef accretion is compromised [4], as are the ecosystem services provided by the reef [5].

Local impacts associated with nutrient enrichment, pollution and overfishing have also increased in the last decades, leading to so called “phase shifts” in many parts of the Caribbean and coral reefs worldwide [6, 7]. One of the main drivers of “phase shifts” is related to inorganic and organic nutrient inputs derived from untreated or poorly treated sewage. The impact of elevated DOC concentrations on coral reef health is currently of major concern in coral reef research [8–10], as elevated DOC has been associated with enhanced bacterial growth and other processes that lead to oxygen depletion and the accumulation of toxic substances, and ultimately to an increase in coral mortality [9, 11, 12]. High concentrations of DOC, predominantly in the form of dissolved carbohydrates, can also enter the coral reef system in the form of exudates released by the benthic community [13, 14]. Previous results have shown minimal or no significant differences in the DOC concentrations released by benthic calcifying algae (*Halimeda opuntia)* compared to coral exudates (*Porites lobata*) [10, 15], although it has been postulated that bacterial growth is primarily triggered by algal-derived DOC rather than DOC released by corals [10, 15].

The sandy bottom of Caribbean reef lagoons are commonly colonized by rhizophytic calcareous green algae (Siphonales) of the genera *Halimeda*, *Udotea*, *Penicillus* and *Rhipocephalus*, which are associated with the seagrass habitat builder *Thalassia testudinum* [16–18]. Calcareous green algae produce an important fraction of coral reef carbonate production in the form of calcareous sand, essential to support reef accretion [19–22]. Most of the studies that have investigated the responses of marine macrophytes to OA and other local threats have focused on species of the genus *Halimeda*, due to this genus is considered one of the most productive. Limited attention has been given to other important reef calcifiers, such as species from the genus *Udotea*, *Penicillus*, *Rhipocephalus*. For experimental studies focused on *Halimeda* spp., it has been concluded that this genus displays a large species-specific variation to increasing levels of dissolved inorganic carbon (DIC). Some species reduce their photosynthetic rates [23, 24], while others have shown positive [25] or no effect on algal photosynthesis [26–28]. Similarly, large inter-specific variation has been documented for the response of calcification of *Halimeda spp*. to DIC increases [24, 26–33] indicating that some species may be more tolerant to OA than others. Altered skeletal structure of different *Halimeda* spp. in response to OA conditions has also been reported [34, 35], being indicative of potential needle dissolution [29] and/or the formation of more slender crystals during exposure to reduced pH [25]. Yet, alteration of skeletal structure may also affect the contribution of species from the genus *Halimeda* to sediment carbonate production under different OA scenarios, irrespective of the severity of the impact detected on algal physiology.

In contrast to OA, nutrient enrichment enhances *Halimeda* spp. production and growth [23, 36, 37], with the exception of phosphate enrichment, for which a large species-specific variation has been also reported [36]. An analysis on the combined effect of inorganic nutrient enrichment and reduced pH on *Halimeda opuntia* has shown decreased enhancement in algal production under nutrient enrichment and reduced pH, relative to the estimated values for ambient pH [38]. Meyer et al. [28] have recently shown the negative effects of increased DOC concentration on the photosynthesis of two *Halimeda* species from the Great Barrier Reef, *H*. *opuntia* and *H*. *macroloba*, but no effect was found on algal calcification rates under illumination. These authors further investigated the combined effect of elevated DOC and DIC concentrations, and documented an adverse impact on thallus photosynthesis for both species, while only *H*. *opuntia* showed a negative effect on dark calcification rates. These findings support the large species-specific component of the response of marine algae to the combined effects of OA and increased DOC, and the importance of increasing the number of this type of experimental studies for other sites and species.

*Halimeda incrassata* (J. Ellis) J.V. Lamouroux and *Udotea flabellum* (J. Ellis & Solander) M. Howe are two abundant rhizophytic species of the macrophyte community of shallow seagrass habitats dominated by the species *Thalassia testudinum*. In the Puerto Morelos reef lagoon, Mexican Caribbean, both species are among the most abundant calcareous algae. Estimates of *H*. *incrassata* primary production for this lagoon (0.2–0.5 g dwt m^-2^ day^-1^) [22] are lower than the reported values for seagrass leaf production (0.9–1.2 g dwt m^-2^ day^-1^) [39]. Interestingly, annual carbonate production for *H*. *incrassata* from this area is in the same range or even lower (between 0.5 and 1.0 kg CaCO_3_ m^-2^ y^-1^) [22] than estimates of annual carbonate production recently documented for the dominant seagrass *T*. *testudinum* (between 0.5 and 5.63 kg CaCO_3_ m^-2^ y^-1^) by Enríquez and Schubert [40]. To our knowledge, no estimates for *Udotea* spp. annual carbonate production are yet available for this area or other locations. The only study on the daily carbonate production of *U*. *flabellum* [41] has documented that this species produces about 45% less carbonate per day than *H*. *incrassata*.

The Mexican Caribbean (Cancún and the Riviera Maya) has experienced a 4.3-fold population increase during the period from 2000–2009 [18], which is associated with the rapid coastal development of large tourist complexes. These changes have severely affected the reef habitat, particularly the benthic macrophyte community associated with seagrass beds, which is shifting towards an increased presence of fleshy macroalgae [18], and increased biomass of green calcifiers [42]. To understand the combined effect of these local impacts with the predicted negative effect of OA on marine calcifiers, this study investigated the direct and combined effects of experimental increases in DIC and DOC on the physiological performance of *Halimeda incrassata* and *Udotea flabellum*. This multi-factorial study aims to analyze a more realistic scenario that may be also very useful for other areas affected by similar coastal eutrophication derived from different anthropogenic impacts. As calcareous macroalgae are considered important contributors to reef carbonate budgets, this study can also contribute to improve our understanding of future impacts caused by the combined effects of global and local threats on reef accretion and the stability of the reef system.

## Materials and Methods

### Algal Collection and Maintenance

Several individuals of *H*. *incrassata* and *U*. *flabellum* were collected by SCUBA diving from the Puerto Morelos reef lagoon, Mexican Caribbean (20° 52’ N, 86° 52’ W), in March 2012 at 3–3.5 m depth, and transported in mesh-covered ziplog bags to the mesocosm facilities of the Universidad Nacional Autónoma de México (UNAM). To minimize physiological variability among replicates associated with age and photoacclimatory condition of the thallus, thalli of similar size and position were selected for the experimental analysis: 5–7 apical segments for *H*. *incrassata*, and *U*. *flabellum* individuals of 3–5 cm height.

The selected individuals were acclimated for 5 days to the experimental conditions by placing them in 50 L experimental tanks receiving filtered (~50 μm) ambient seawater (~28°C, pH 8.2) from the lagoon, with continuous flow of 1 L min^-1^. Irradiance levels at mesocosms were adjusted using neutral density shade mesh to simulate light conditions at collection depth (51% of surface irradiance, E_s_). E_s_ was calculated using surface irradiance data and the down-welling light attenuation coefficient of the reef lagoon estimated for the sampling period (February-March 2012) of K_d_ = 0.2 m^-1^, which was similar to previous values reported [43]. Variation in diurnal irradiance was continuously recorded throughout the experiment using a cosine-corrected light sensor (LI-190SA; LI-COR, Lincoln, NE, USA) connected to a data logger (LI-1400; LI-COR, Lincoln, NE, USA), located at the mesocosm system.

After the pre-acclimation period (5 days), initial measurements of photosynthesis, respiration and calcification rates were performed (n = 6) as described below, and 12 individuals of each species (n = 12) were randomly positioned into each tank.

### Experimental Treatments

The experiment was conducted over 10 days in an open flow-through system, which consisted of 12 tanks of 50 L each. To enhance water movement in the tanks and prevent carbon limitation of algal photosynthesis, aquaria pumps (500 L h^-1^) were located in each tank and connected to a rectangular PVC frame surrounding the tank with holes facing inward to create homogenous flow conditions according to Cayabyab and Enríquez [44]. We used three replicate tanks per treatment and placed tanks with the different treatment levels (ambient and increased, see below) in alternating order.

The treatments comprised ambient and elevated *p*CO_2_ concentrations in order to simulate OA changes in CO_2_ availability from 380 to 1000 μatm, respectively, as well as ambient and increased DOC conditions (see Table 1). The increase in *p*CO_2_ concentration was achieved by pH manipulation via CO_2_ gas injection by a potentiometric pH sensor controlled pH stat system (IKS Aquaristic Products, Karlsbad, Germany). pH-reading by the pH-stat system was continuous (every other second) to adjust pH levels in the system, and pH sensors were calibrated every other day according to values measured by a WTW Multi 3430 probe (WTW, Weilheim, Germany). pH in the tanks was maintained at 8.2 for the control treatment, and reduced to 7.8 for the high DIC treatments (see Table 1). The elevated *p*CO_2_ levels used here were selected considering the Representative Concentration Pathways 8.5 (RCP8.5), which predicts a decrease of seawater pH between 7.7–7.8 [45].

**Table 1:** Variation in the experimental conditions. Values for the carbonate system parameters were calculated using CO2SYS with temperature, salinity, total alkalinity (TA) and pH_NBS_ as input parameters (n = 3). Additionally, the Biological Oxygen Demand (BOD) of the seawater in each treatment are given, determined through oxygen consumption using dark incubations over 24 h and referring the estimated changes to the water volume of the incubation (n = 3). Data represent mean ± SD (n = 6) and the results of a one-way ANOVA (p<0.05) performed to determine significant differences in BOD between treatments, are indicated by different letters.

Treatment	pH [NBS]	DOC (μmol L^-1^)	T°C	Salinity (ppt)	TA (μmol kgSW^-1^)	*p*CO_2_ (μatm)	HCO_3_ (μmolkg SW^-1^)	Ω_Ar_	BOD (mg O_2_ L^-1^ h^-1^)
Control	8.22 ±0.02	171	28.1±0.1	35.9 ±0.1	2414 ±8	377 ±27	1776 ±26	4.2 ±0.2	0.63 ±0.6^a^
High DIC	7.84 ±0.04	171	28.0±0.2	36 ±0.1	2409 ±6	1076 ±101	2097 ±24	2.0 ±0.1	1.03 ±0.3^a^
High DOC	8.19 ±0.03	550	28.1±0.2	35.9 ±0.1	2414 ±8	415 ±31	1811 ±31	4.0 ±0.2	1.35 ±0.3^a^
High DOC & DIC	7.82 ±0.02	550	28.0±0.1	36 ±0.1	2413 ±7	1135 ±55	2114 ±14	2.0 ±0.1	1.62 ±0.4^a^

**Fig 1:**
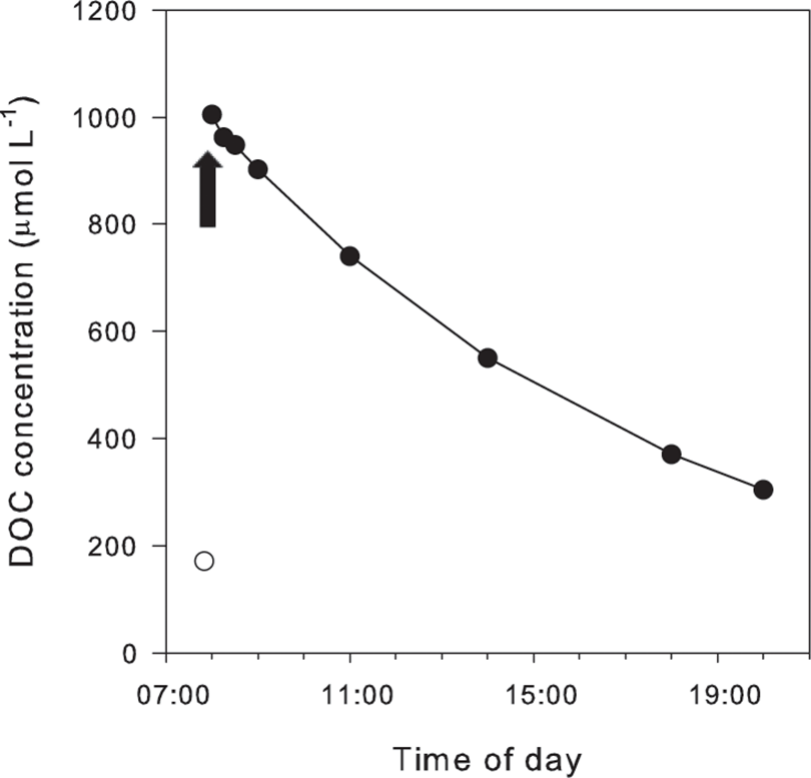
DOC concentrations in the high DOC-treatments measured during a 12-hour cycle, from 8:00 a.m. to 20:00 pm, after addition of 833 μmol L^-1^ DOC as glucose (indicated by an arrow). Filled-solid circles represent the average of two sampling points (n = 2) of the high DOC-treatments, and the open-white circle indicates the ambient DOC concentration in the non-DOC-enriched treatments.

DOC treatment levels were adjusted to concentrations described in previous studies on coral communities, using glucose and lactose in high concentrations as DOC [8, 9, 46]. In this study, the DOC treatment in the form of highly bioavailable DOC was achieved by additions of 833 μmol L^-1^ DOC (Glucose, D-Glucose, Sigma Aldrich) twice daily at 08:00 and 20:00 to each of the six high-DOC treatment tanks, simulating sudden DOC enrichments events, common in nature associated with strong rain. To quantify the resulting DOC treatment conditions, DOC concentration were measured over a 12-hour cycle (Fig 1), showing an average DOC concentration of 550 μmol L^-1^ (Fig 1). Samples for TOC were filtered through 0.45 μm GFF filters (Whatman), acidified with 150 μL fuming HCl and frozen at -20°C until analysis using a Shimadzu TOC-5000A (Shimadzu, USA).

Salinity and temperature were also monitored for each tank twice daily throughout the experiment (WTW Multiprobe 3430), and total alkalinity (TA) every second day. Water samples from the tanks were filtered through 0.45 μm GFF filters and stored with a drop of chloroform at 4°C until TA analysis. These parameters, together with the pH values on the NBS scale taken every two days with a multiprobe (WTW3430, Weilheim, Germany), were used to calculate the carbonate system using the CO2SYS excel spreadsheet software, with the constants from Mehrbach et al. [47] (Table 1).

### Biological Oxygen Demand (BOD)

To evaluate potential enhancements in microbial respiration rates in seawater, BOD was measured at the end of the experiment. We incubated 150 mL of unfiltered seawater in Winkler bottles for 24 h in the dark (n = 3), under constant temperature conditions (28°C). Oxygen concentration (mg L^-1^ and % saturation), as well as salinity and temperature were also measured before and after incubations. O_2_ consumption rates in mg O_2_ L^-1^ h^-1^ were calculated and corrected for water volume and length of incubation.

### Assessment of Maximum Photosynthetic Quantum Efficiency

Maximum photochemical efficiency of photosystem II—PSII (F_v_/F_m_) of experimental organisms was measured every evening at 20:00 on the apical segments of the organisms, using a Pulse Amplitude Modulated fluorometer (Diving-PAM, Walz, Germany). At this time, one hour after sunset, algal thalli had already achieved the maximum F_v_/F_m_ of the day, as all the non-photochemical quenching processes were relaxed, and the maximum PSII recovery of the day had been already reached (see [44, 48]).

### Quantification of Photosynthesis, Respiration and Light Calcification Rates

For physiological measurements, young 4–5 apical segments of *H*. *incrassata* thalli and the uppermost 2 cm of *U*. *flabellum* thalli were selected (two organisms per tank and species) to reduce the variation among replicates in the thallus physiological condition, due to age, photoacclimation, abundance of epiphytes and/or accumulation of damage. The segments were separated from the parent plant at least 2 h before physiological determinations were started in order to allow complete wound healing [49].

Before and at the end of the experiment, photosynthesis and calcification rates were simultaneously determined by incubating algal thalli for 30 min under a saturating light intensity of 500 μmol quanta m^-2^ s^-1^ (three times the E_k_ of the species, data not shown), in freshly filtered seawater obtained from the respective treatment tanks. The incubation water (17 mL) was collected at the beginning and at the end of the light incubation to determine the alkalinity changes induced by algal activity (see below). The samples were incubated in darkness for another 10–15 min to determine the post-illuminatory respiration rate (R_L_). Oxygen evolution rates were measured polarographically in water-jacked chambers (DW3, Hansatech Instruments Ltd., Norfolk, UK), using Clark-type O_2_ electrodes (Hansatech). A circulating bath with a controlled temperature system (RTE-100/RTE 101LP; Neslab Instruments Inc., Portsmouth, NH, USA) allowed maintenance of a constant temperature of 28°C (treatment temperature) during the incubation. The electrodes were calibrated with air- and N_2_-saturated filtered seawater. Freshly filtered seawater (0.45 μm) from the respective treatment tank was used for the incubations, with DIC and DOC concentrations corresponding to the treatment conditions (see Table 1). Data were captured with a computer equipped with an analog/digital converter using DATACAN V software (Sable Systems, Inc., Las Vegas, NV, USA). Gross photosynthesis was calculated adding to the net photosynthesis determined in the incubations, the oxygen consumption through post-illuminatory respiration.

Calcification rates were determined using the alkalinity anomaly principle based on the ratio of two equivalents of total alkalinity for each mole of precipitated CaCO_3_ [50]. For alkalinity measurements, a modified spectrophotometer procedure as described by [51] and [40] was used. For quality control, a certified reference material of known total alkalinity (CRM, Scripps Institution of Oceanography, USA) was used to calibrate the method.

### Quantification of Algal Surface Area

For normalization of the measured metabolic rates, the surface area of each algal segment was determined by scanning the thalli and analyzing the digital images using ImageJ software.

### Statistical Analyses

Data were tested for normality using the Shapiro-Wilk test, and for equal variance using the Levene median test. Analyses of variance (ANOVA) allowed for the determination of significant differences (*p*<0.05) between the different descriptors used to characterize the physiological response of the species. A one-way ANOVA was used to compare initial photosynthetic, respiratory and calcifications rates; the calcification / photosynthesis ratio; and for the comparison of BOD values between each treatment and the control. A t-student test was used to evaluate significant differences between initial and final F_v_/F_m_ values with respect to the control organisms. To analyze whether F_v_/F_m_, photosynthesis, respiration and calcification rates differed significantly between treatments, two-way-ANOVA tests were used, considering the DIC and DOC treatments as fixed factors to test for direct effects, as well as the interaction (DIC x DOC). For the comparison of differences between individuals and treatment combinations, a Newman-Keuls Post-hoc test was used. The statistical analyses were conducted using Statistica 12.0.

## Results

*Halimeda incrassata* showed significantly higher photosynthetic and calcification rates than *Udotea flabellum*, with no difference in post-illuminatory respiratory rates (Table 2). These differences were also reflected in their calcification to photosynthesis ratios. While *H*. *incrassata* was able to precipitate 0.57 (±0.08) mol CaCO_3_ per mol O_2_ evolved in photosynthesis, *U*. *flabellum* (ANOVA, *p* = 0.038) only precipitates 0.25 (±0.07) mol CaCO_3_ per mol O_2_ produced (Table 2).

**Table 2:** Comparison of the initial values (day 0) of gross maximum photosynthetic rates (P_max_), post-illumination respiration (R_L_), maximum calcification rates (G_max_), and the ratio of calcification:photosynthesis (G_max_:P_max_) of *Halimeda incrassata* and *Udotea flabellum*. Data represent mean ± SE (n = 6) and significant differences between species (one-way ANOVA, p<0.05) are indicated by different letters.

Metabolic rates	*Halimeda incrassata*	*Udotea flabellum*
P_max_	2.33±0.08^a^	1.56±0.06^b^
R_L_	0.39±0.02^a^	0.47±0.05^a^
G_max_	1.32±0.2^a^	0.40±0.16^b^
G_max_:P_max_	0.57±0.08^a^	0.25±0.07^b^

**Fig 2:**
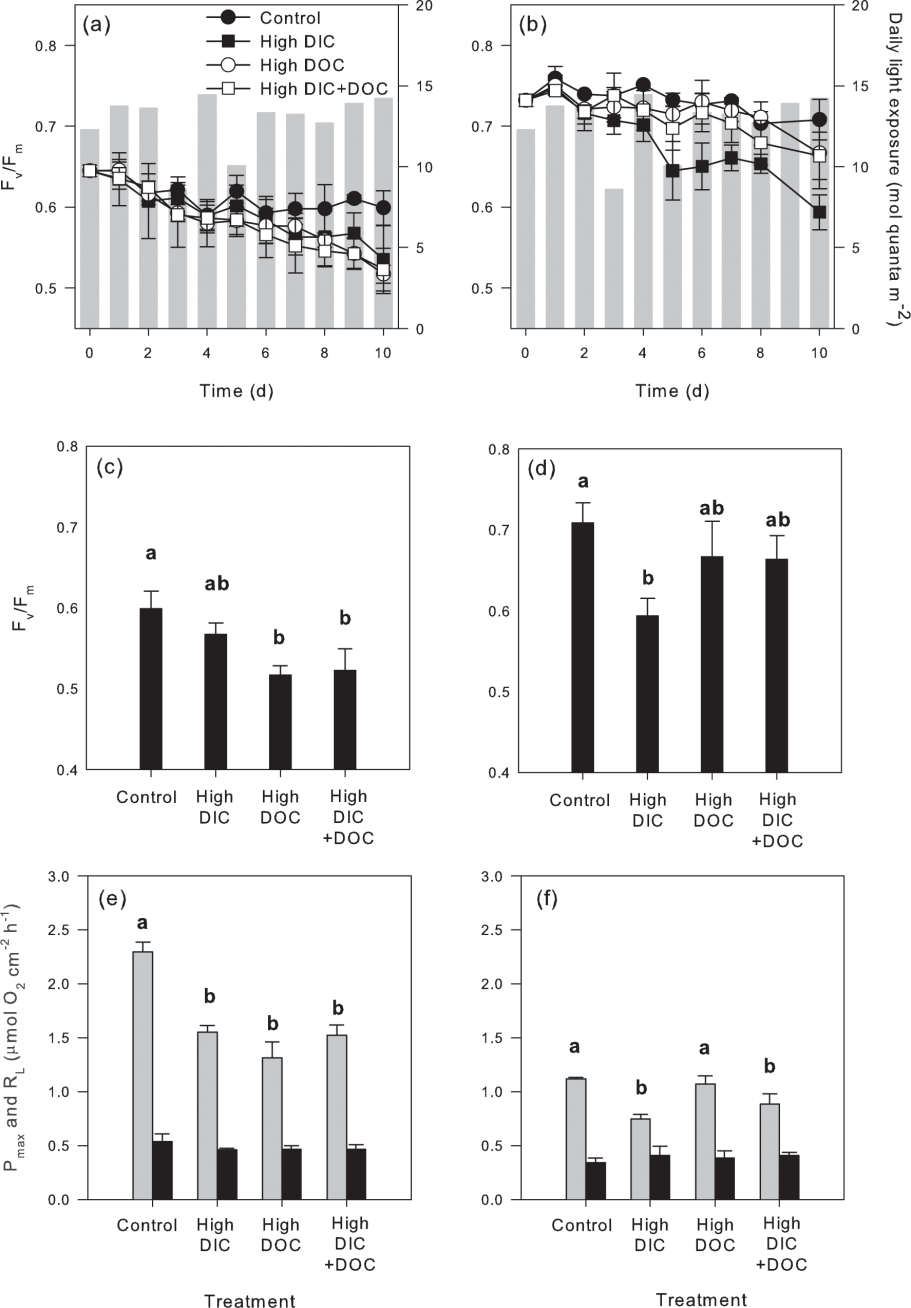
Photosynthetic responses of *H*. *incrassata* (a, c, e) and *U*. *flabellum* (b, d, f) to the experimental DIC and DOC treatments. (a, b) Daily variation in the maximum quantum efficiency of photosystem II (F_v_/F_m_) along the experiment and the corresponding daily integrated values in light exposure in mol quanta m^-2^ day^-1^ (grey bars); (c, d) F_v_/F_m_ for each treatment at the end of the experiment; and (e, f) gross photosynthesis, P_max_, (light grey bars) and respiration, R_L_, (black bars) rates at the end of the experiment. Data represent mean ± SE (n = 3) and significant differences between treatments (ANOVA, Newman-Keuls, p<0.05) are indicated by different superscript letters.

In addition, the two species showed contrasting responses to experimental DIC and DOC treatments. The response of maximum photosynthetic rates was similar in both species, but more pronounced than indicated by the F_v_/F_m_ response. The variation in F_v_/F_m_ was closely related to the diurnal variation in solar radiation. Control organisms showed a similar pattern of variation in both species, with a slight, but non-significant decline over time (t-test, *p* = 0.528 for *H*. *incrassata*, *p* = 0.560 for *U*. *flabellum*) compared to initial values (Fig 2A and 2B). When comparing final F_v_/F_m_ values, *H*. *incrassata* showed a significant decline in the DOC treatments, under both ambient and elevated DIC concentrations (Fig 2C, Table 3), while *U*. *flabellum* only showed a negative response of F_v_/F_m_ under elevated DIC (Fig 2D, Table 2). Significant reductions in P_max_ were estimated for *H*. *incrassata* in all treatments, when compared to control organisms. P_max_ reductions ranged from -30% (elevated DIC) to -43% (elevated DOC; Fig 2E), and showed a significant effect in the combined treatment (Table 3). In contrast, *U*. *flabellum* experienced a significant reduction in P_max_ under elevated DIC compared to the control (high DIC: -33%; high DIC+high DOC: -21%), while elevated DOC did not cause any effect on thallus photosynthesis (Fig 2F, Table 3). Thallus respiratory rates were not affected by any experimental treatment in any species (Fig 2E and 2F).

**Table 3:** Two-way ANOVA analyses performed to determine significant differences in the physiological responses of the apical segments of *Halimeda incrassata* and *Udotea flabellum* exposed to four experimental treatments: control, high DIC concentration, high DOC concentration, and the combined treatment (n = 3 for each treatment). **DIC and DOC were considered fixed factors and DIC x DOC show the interaction between both factors**.

Response variable	Species	Source of variation	DF	SS	MS	F-value	*p*-value
Maximum quantum yield (F_v_/F_m_)	*H*. *incrassata*	DIC	1	0.0005	0.0005	0.69	0.4275
		DOC	1	0.0121	0.0121	16.36	0.0037*
		DIC x DOC	1	0.0011	0.0011	1.45	0.2632
		Residual	8	0.0059	0.0007		
	*U*. *flabellum*	DIC	1	0.0104	0.0104	5.85	0.0419*
		DOC	1	0.0006	0.0006	0.33	0.5790
		DIC x DOC	1	0.0093	0.0093	5.21	0.0519
		Residual	8	0.0142	0.0018		
Gross	*H*. *incrassata*	DIC	1	0.162	0.162	6.267	0.0368*
Photosynthesis (P_max)_		DOC	1	0.863	0.863	33.37	0.00042*
		DIC x DOC	1	0.590	0.590	22.82	0.00140*
		Residual	8	0.207	0.026		
	*U*. *flabellum*	DIC	1	0.232	0.232	25.96	0.0009*
		DOC	1	0.006	0.006	0.68	0.4349
		DIC x DOC	1	0.027	0.027	2.99	0.1222
		Residual	8	0.071	0.009		
Respiration (R_L_)	*H*. *incrassata*	DIC	1	0.0043	0.0043	1.10	0.3253
		DOC	1	0.0039	0.0039	0.98	0.3506
		DIC x DOC	1	0.0048	0.0048	1.21	0.3034
		Residual	8	0.0316	0.0039		
	*U*. *flabellum*	DIC	1	0.0061	0.0061	1.14	0.3171
		DOC	1	0.0016	0.0016	0.30	0.5987
		DIC x DOC	1	0.0010	0.0010	0.19	0.6689
		Residual	8	0.0425	0.0053		
Light calcification (G_max_)	*H*. *incrassata*	DIC	1	1.16	1.16	23.33	0.0013*
		DOC	1	0.495	0.495	9.98	0.0134*
		DIC x DOC	1	4.38	4.38	88.28	0.000013*
		Residual	8	0.397	0.0496		
	*U*. *flabellum*	DIC	1	0.018	0.018	6.184	0.0378*
		DOC	1	0.399	0.399	140.94	0.000002*
		DIC x DOC	1	0.002	0.002	0.778	0.4034
		Residual	8	0.023	0.0028		

**Significant results (p<0.05) are marked in bold and** with an asterisk (*).

**Fig 3:**
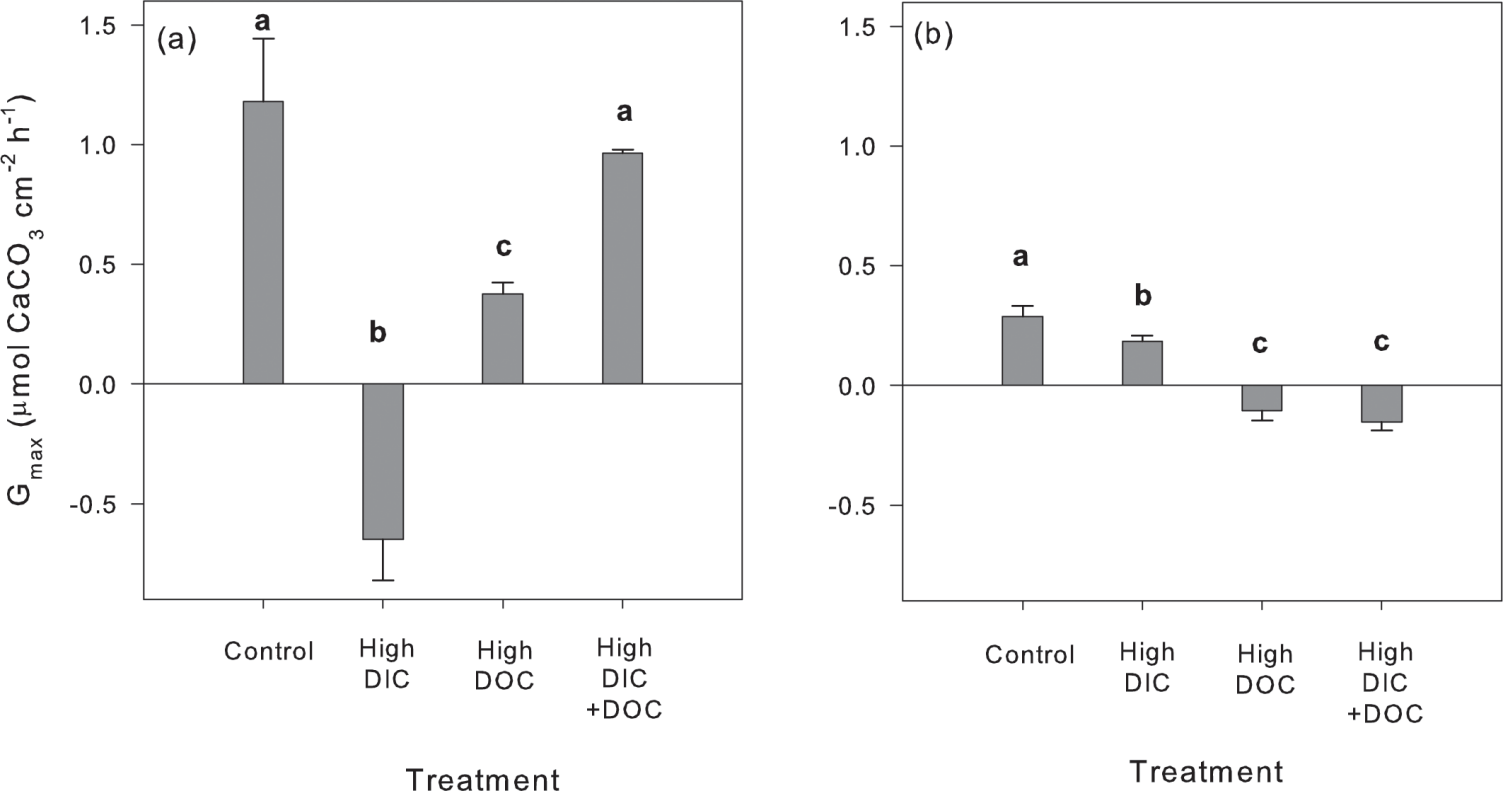
Calcification (G_max_) response of *H*. *incrassata* (a) and *U*. *flabellum* (b) to the experimental DIC and DOC treatments. Data represent mean ± SE (n = 3) and significant differences between treatments (ANOVA, Newman-Keuls, p<0.05) are indicated by different letters.

The response of thallus calcification to the experimental treatments also showed large differences between species. While *H*. *incrassata* showed full suppression of thallus calcification and even dissolution of CaCO_3_ after exposure to elevated DIC, thallus calcification was still positive albeit significantly reduced in *U*. *flabellum* after exposure to the same treatment (-36% compared to control; Fig 3). The opposite response was observed for elevated DOC, as we found a significant decline in calcification rates of *H*. *incrassata* (-68%) with respect to control organisms (yet positive values), while no calcification but dissolution of CaCO_3_ (negative values) was measured for *U*. *flabellum* (Fig 3). The inhibition of thallus calcification by elevated DOC concentration was further exacerbated in *U*. *flabellum* in the combined treatment, due to the addition of the negative effect of DIC (Fig 3B), as no significant interactive effect was found for the response of thallus calcification in this species (Table 3). In contrast, the combined treatment did not show any significant impact on *H*. *incrassata* calcification, notwithstanding the significant negative direct effects of elevated DOC and DIC (Fig 3A). These findings support the antagonistic effect between elevated DIC and DOC and their combined effect on the calcification process of *H*. *incrassata* (Table 3).

Additionally to the physiological responses of the organisms, measurements of BOD for the different treatment waters were performed to determine potential changes in bacterial respiration. Although increased BOD was detected in the treatments with elevated DOC, these changes were not significant (Table 1).

## Discussion

Large differences between the two species investigated were found in photosynthesis and calcification rates, in agreement with previous findings [41]. Our study further revealed significant differences between *Halimeda incrassata* and *Udotea flabellum* in the calcification:photosynthesis ratio (G_max_/P_max_), as *H*. *incrassata* was able to precipitate twice as much CaCO_3_ per mol O_2_ evolved in photosynthesis than *U*. *flabellum*.

Elevated DIC and DOC treatments caused adverse impacts on the physiology of both species, but significant differences were observed in the severity of this impact. For example, elevated DIC resulted in a decline in F_v_/F_m_ and photosynthesis rates in both species. However, elevated DOC only caused a similar response in *H*. *incrassata*, as non-significant changes were observed for the organisms of *U*. *flabellum* exposed to the similar treatment. Control organisms did not show the progressive reduction in F_v_/F_m_ observed for organisms exposed to DIC and DOC enrichments throughout the experiment. This lack of change in F_v_/F_m_ after an initial reduction during the first four days, in spite of the maintenance of high light conditions for the last five experimental days, indicates that experimental conditions were optimal for both species and did not induce significant accumulation of photodamage (i.e., F_v_/F_m_ decline), or positive F_v_/F_m_ recovery due to light limitation. Thus, the observed reductions in F_v_/F_m_ and thallus photosynthesis of the organisms exposed to elevated DOC and/or DIC can be attributed to a direct negative impact of these treatments on the photosynthetic process. Thallus photosynthesis in *U*. *flabellum* showed a more robust response to elevated DOC, while *H*. *incrassata* was equally sensitive to both organic and inorganic carbon enrichments. A similar negative impact of elevated DIC on algal photosynthesis has been previously reported for other species from the genus *Halimeda* [26, 29, 30], but the causes of this decline have not yet been elucidated. Price et al. [26] suggested that the increase in dissolved CO_2_ under reduced seawater pH may affect the expression of different carbon-concentrating mechanisms (CCMs), causing algal photosynthesis to rely on passive CO_2_ diffusion, and thus becoming more susceptible to photosynthesis carbon limitation. The maintenance of high proton-H^+^ permeability of the plasma membrane, for example, which is key for photosynthetic bicarbonate assimilation [52, 53], declines at reduced external pH [54].

In addition to the impact of DIC on algal photosynthesis, we also found a negative effect of elevated DIC on calcification rates of both species. *Udotea flabellum* showed a similar -30% reduction in photosynthesis and calcification (-36%; Figs 2F and 3B), but *H*. *incrassata* experienced larger declines in calcification (-155%) compared to a -30% reduction in photosynthesis (Figs 3A and 2E). With respect to the response to elevated DOC concentration, a greater impact was observed on *H*. *incrassata* photosynthesis and calcification rates when acting in isolation. Negative effects of elevated DOC concentrations have been recently documented for the photosynthesis rates of two *Halimeda* species from the Great Barrier Reef [28]. In contrast to our findings, calcification under illumination was not significantly affected by elevated DOC in these species. Large differences for the response of thallus calcification to elevated DIC have been already documented among *Halimeda* spp. [26, 29, 30], and this is the first time that similar inter-specific differences were also observed for the response to elevated DOC. Some authors have suggested that the large inter-specific component shown by the calcification process in the genus *Halimeda* may rely on thallus morphology [26]. This genus displays large variation in the internal anatomy of algal thallus, and these anatomical characteristics are good proxies for species membership when compared to molecular data [55], what may support our interpretation. However, more work is still needed to elucidate the potential implications of the variation in thallus anatomy within the *Halimeda* genus on the species-specific sensitivity of thallus calcification to environmental changes.

Photosynthesis and calcification rates are tightly coupled in calcareous siphonal algae. Photosynthesis promotes algal calcification by removing CO_2_ or bicarbonate from the calcification site, which increases the local pH and, thus, facilitates CaCO_3_ precipitation [56]. Photosynthesis can also support a high fraction of the energetic costs of the biomineralization process. Therefore, any negative effect on the photosynthetic process would be reflected in a decline in algal calcification, as recently shown for coralline algae [48]. Inter-specific differences in the calcification process may explain the diversity of responses observed. For example, while *H*. *incrassata* only calcifies in the intercellular spaces, calcification in *U*. *flabellum* represents a transition between intercellular and sheath mineralization (e.g. *Penicillus*, *Rhipocephalus*) [57]. The CaCO_3_ precipitation in *H*. *incrassata* occurs in a semi-isolated space, where CO_2_ diffusion from the external environment can cause a decrease in local pH, and thus a reduction in calcification rates. Therefore, a more efficient isolation from surrounding seawater of the biomineralization site of *U*. *flabellum*, allows carbonate precipitation to be less dependent on the external variation of DIC and, thus, better suitable to be controlled by the physiology of the organism. The occurrence of a stronger control over the CaO_3_ precipitation process by *U*. *flabellum* is supported by the findings of Ries [41].

Calcareous green algae are able to release DOC, but cannot incorporate organic carbon [58–59]. Thus, although DOC decline in the enriched experimental treatments was primarily due to water turnover rates in the tanks, part of this DOC enrichment could have likely been assimilated by bacteria (Fig 1). Furthermore, the experimental addition of DOC in the form of glucose stimulates microbial respiration and growth [60]. Such enhancement in bacterial activity explains the lower O_2_ concentrations observed in DOC-treatments compared to control- and DIC-treatments, as reported previously [46, 61]. Little information is available about the interaction between these epibacterial communities and algal physiology, and the potential effects of environmental changes on these communities and their interactions (i.e., [62–64]). It has been documented for *Halimeda copiosa* that the abundance of thallus surface-associated bacteria increases under organic nutrient enrichments [65]. In addition to increases in bacterial abundance, shifts in the bacterial community towards non-beneficial or even harmful bacteria have been suggested to occur for corals under increasing DOC concentrations [8]. As no increases in algal respiratory rates were observed in the DOC-treatments (Fig 2E and 2F), the negative responses on photosynthetic and calcification rates were most likely related to alterations in the bacterial community than in their abundance. Benthic reef algae have been shown to differ in the microbial communities associated with their tissue [66], therefore, part of the observed differences in the DOC response of *H*. *incrassata* and *U*. *flabellum* might be related to differences in the response of their respective epibacterial communities to the experimental DOC enrichment, as well as species-specific effects on bacterial-algal interactions. The antagonistic effect found for the combined elevated DIC and DOC treatment in *H*. *incrassata*, could also be due to a differential effect of each factor on the bacterial-algal interactions. More studies focusing on the seaweed holobiont are necessary to fully understand the relevance of these indirect effects on algal performance.

### Ecological Perspective

According to our results, the DIC concentrations expected by the year 2100 [45] may significantly reduce photosynthesis and carbonate production in *H*. *incrassata*, while *U*. *flabellum* production may experience relative lower declines. However, when accompanied by increased concentrations of high labile DOC, the impact of elevated DIC will be alleviated for *H*. *incrassata* but exacerbated for *U*. *flabellum*. Furthermore, the effect of DIC and DOC could be even more severe when considering their impact at night on algal calcification, as has been documented for dark calcification rates that are also negatively affected in *Halimeda* spp. [28, 67]. Thus, considering the impacts both during the light and night on thallus calcification rates, the net algal carbonate production can be reduced even further.

Potential sources of high labile DOC for this particular reef lagoon are the seagrass and macroalgal beds themselves [59], human waste water discharge via groundwater [68, 69], and storm events [70], which are all predicted to increase in the future providing more labile DOC to this coastal ecosystem. For the carbon budget of the Puerto Morelos reef lagoon [71], this DOC enrichment may lead to a significant reduction of the contribution of calcifying green algae to the overall primary production and/or carbonate reef accretion. This impact will produce severe consequences on the macrophyte community, habitat structure, and ultimately, on the organic carbon fluxes of the ecosystem due to altered contributions to labile DOC and POC pools [59]. Significant reductions in carbonate sand production from algal derived sediments can alter the volume of sand deposits in coastal tropical areas, with important consequences for beach erosion and coastal sediment dynamics of reef environments. On the other hand, considering that seagrasses and fleshy algae may prosper under higher DIC conditions [72–74], and that seagrasses can modulate the OA response of calcareous algae [75–77], a deeper understanding of the changes in the macrophyte community and on species interactions will be fundamental to enhance our capacity to foresee the severity of the impact of predicted environmental changes on carbonate sand production by calcareous green algae.
